# Embryonal Rhabdomyosarcoma - A Mimicker of Squamosal Otitis Media

**DOI:** 10.22038/ijorl.2019.38807.2280

**Published:** 2020-01

**Authors:** Ajay Bhandarkar, Architha Menon, Ranjini Kudva, Kailesh Pujary

**Affiliations:** 1 *Department of * *Otorhinolaryngology* *, Kasturba Medical College, Manipal Academy of Higher Education Madhavnagar Manipal - 576104.*; 2 *Department of Pathology, Kasturba Medical College, Manipal Academy of Higher Education Manipal.*

**Keywords:** Acute facial paralysis, Chemotherapy, Pediatric, Rhabdomyosarcoma, Radiotherapy, Temporal Bone

## Abstract

**Introduction:**

Rhabdomyosarcoma is the most frequently occurring intrusive soft tissue sarcoma in the pediatric age group. Orbit is the most common location for a pediatric rhabdomyosarcoma, but it can occur in the oral cavity, pharynx, face and neck in the descending order of incidence. Rhabdomyosarcoma in the ear is extremely rare.

**Case Report:**

A 5-year-old girl presented to the outpatient department of our tertiary care hospital with complaints of foul smelling, non-blood stained right ear discharge of one-month duration and deviation of angle of mouth to the left side of acute onset. Investigations revealed a diagnosis of embryonal rhabdomyosarcoma. Multimodal therapy was carried out, and the child was rendered disease-free after two years.

**Conclusion:**

Embryonal rhabdomyosarcoma of the head and neck mimics chronic otitis media. Early diagnosis is essential to deliver prompt treatment and prevent locoregional spread and metastasis.

## Introduction

Rhabdomyosarcoma is the most frequently occurring intrusive soft tissue sarcoma in the pediatric age group (1-3). It is the third most common tumor in childhood (2). Origin of rhabdomyosarcoma in the head and neck account for approximately 35% of all pediatric rhabdomyosarcoma and has an incidence of 4.6 per million in the pediatric age group (2-4).

Orbit is the most common location for a pediatric rhabdomyosarcoma, but it can occur in the oral cavity, pharynx, face and neck in the descending order of incidence (5).

Rhabdomyosarcoma in the ear is extremely rare (4). Diagnosis of rhabdomyosarcoma in the ear is extremely challenging as the symptoms mimic chronic otitis media (3). We present a case of a young girl with acute lower motor neuron facial paralysis and ear discharge. This was in the course of management diagnosed as the embryonal variant of rhabdomyosarcoma. The management of the patient with a review of literature is discussed.

## Case Report

**Fig 1:**
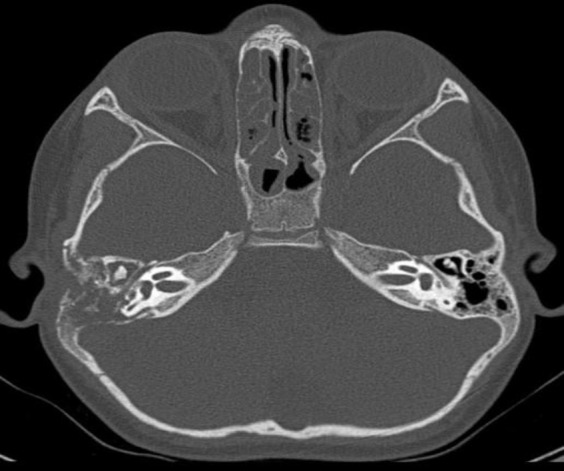
Pre-operative HRCT Temporal Bone showing a soft tissue density in the right external auditory canal and middle ear with erosion of bone

A 5-year-old girl presented to the outpatient department of our tertiary care hospital with complaints of foul smelling, non-blood stained right ear discharge of one-month duration. There was a deviation of angle of mouth to the left side of 15 days duration. She did not have a history of decreased hearing, ear pain, itchiness, tinnitus and vertigo. The child did not have any other neurological deficit. There was no history of fever. On examination of the right ear, a pale polyp was visualised in the right external auditory canal with mucopurulent ear discharge without visualisation of the tympanic membrane. There was House Brackmann Grade 6 lower motor neuron paralysis of the right facial nerve. Rest of the otolaryngological and systemic examination was within normal limits. HRCT of the temporal bone revealed a soft tissue density in the right external auditory canal and middle ear with osteolysis. It also revealed erosion of the horizontal segment of the facial canal with thickening of the nerve distal to the second genu (Fig.1). A provisional diagnosis of chronic otitis media, squamosal type, active stage was made and a differential diagnosis of other erosive disorders of bone like granulomatous diseases and tumors was also considered. Pure tone audiometry revealed right-sided moderate conductive hearing loss. 

**Fig 2:**
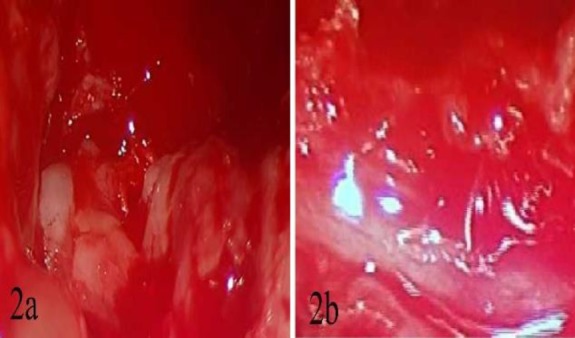
**a.** Microscopic image of a friable mass in the middle ear eroding the facial canal. **b.** Microscopic image of the facial nerve after clearance of mass and facial nerve decompression

The child underwent mastoid exploration under general anaesthesia. Intraoperatively, it was noted to be a friable mass with no evidence of cholesteatoma (Fig.2a). Biopsy from the mass in the middle ear and mastoid was taken. Subsequently, a canal wall down masto- idectomy with facial nerve decompression was done (Fig.2b). 

**Fig 3:**
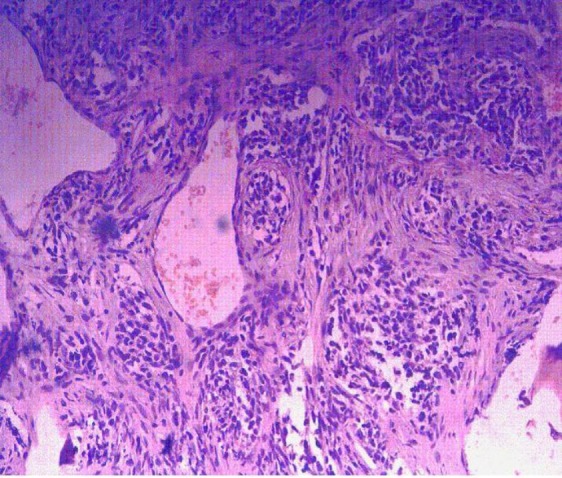
Histopathological Image showing features suggestive of rhabdomyosarcoma

Histopathological examination revealed a subepithelial tumor composed of small round hyperchromatic, pleomorphic nuclei, inconspicuous nucleoli, abundant mitosis and spindle-shaped cells arranged diffusely in a fibromyxoid stroma with congested vessels, areas of haemorrhage and necrosis. Occasional cells with eccentric nuclei and eosinophilic cytoplasm (rhabdomyoblasts) were also visualized (Fig.3). 

**Fig 4:**
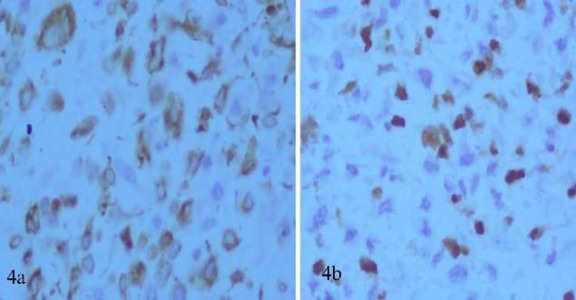
**a. **Immunohistochemistry showing Desmin positivity. **b.** Immunohistochemistry depicting Myogenin positivity

Immunohistochemistry was positive for desmin and myogenin suggestive of embryonal rhabdomyosarcoma (Fig.4a,4b).

The child was advised to undergo PET-CT to evaluate the extent of disease and metastasis. PET-CT revealed low-grade metabolic activity in the right mastoid and petroclival region. There was thrombosis of the right internal jugular vein and adjacent sigmoid sinus without any intracranial extension. 

**Fig 5:**
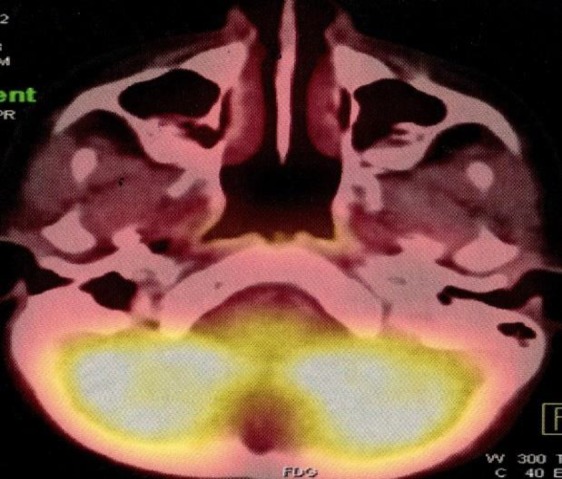
Post-treatment PET-CT image depicting no tumor activity

There was no evidence of distant metastasis. The child underwent 14 cycles of chemotherapy with Vincristine, Actinomycin D and Cyclophosphamide. The initial five cycles of chemotherapy were followed by external beam radiation therapy of 50.4 Gy divided into 28 fractions administered over five and a half weeks. Following the radiation therapy, she underwent nine more cycles of chemotherapy with Vincristine, Actinomycin D and Cyclophosphamide over six months. Facial function improved and was normal six months after therapy. PET-CT done one year following treatment revealed no evidence of residual/recurrent tumor. The patient is on regular follow-up and PET-CT done two years after definitive chemoradiation showed no evidence of tumor (Fig.5). Pure tone audiometry, however, revealed a profound hearing loss and she was suggested a hearing device.

## Discussion

Rhabdomyosarcoma is an aggressive malignant tumor that has a bimodal distribution in children with an incidence reported in the age group of 2-5years and late adolescence. More than 63 percent of tumors occur in the first decade of life (5,6). Literature suggests it forms 5-8% of all pediatric malignancies and 50% of all soft tissue malignant sarcomas (4). Four variants of rhabdomyosarcoma have been reported in literature as per Horn and Enterline classification. They include embryonal, pleomorphic, alveolar and botyroid variants (5).

The embryonal variant is the commonest, whereas, alveolar variant has been known to have the worst prognosis (5,6). The embryonal variant is commonly seen in the head and neck in the first decade of life, whereas, alveolar variant is seen in the extremities in the second and third decades. The pleomorphic variant is seen in the thigh and other skeletal muscle from the fourth decade of life (4).

The symptoms of rhabdomyosarcoma presenting in the ear are similar to chronic otitis media or otitis externa which include blood-stained ear discharge, reduced hearing and ear pain and related complications like facial weakness maybe present. Signs may include polyp or granulation in the external auditory canal (1,3,4,6-8). Clinical progression is rapid from the onset of symptoms to produce facial nerve paralysis and rarely other neurological symptoms and abducent nerve paralysis or headache maybe present due to the invasion of the meningeal barrier (2,4,7). Embryonal rhabdomyosarcoma may spread by hematogenous route, lymphatic route, along the nerve or by the destruction of bone. Middle ear rhabdomyosarcomas have a tendency to invade the facial canal and spread to the internal auditory meatus (9). They may also present with enlarged neck nodes which may show the presence of malignant invasion of tumor in 30% of cases (4,6). Hence, any child with acute onset facial paralysis and symptoms similar to chronic otitis media, squamosal variant should also raise suspicion of rhabdomyosarcoma (5). 

Our patient presented with acute facial palsy and bloodstained ear discharge which mimicked chronic otitis media presentation and eventually proven to be embryonal rhabdomyosarcoma on histopathology. The most common site of distant metastasis is lungs followed by skeletal system, liver and brain (9).

 HRCT, MRI and histopathology with immunohistochemistry form the pillars of diagnosis in rhabdomyosarcoma. HRCT reveals destruction of bone with heterogeneous attenuation within the temporal bone (3,5,7). PET scan will reveal the exact extent of the disease including metastasis and post-treatment recurrence/residual lesion (8). MRI will reveal an expansile, lytic, invasive lesion with low to intermediate signal on T1W and heterogeneous or high signal intensity on T2W (2-4,9). Histopathology shows a round cell tumor and immunohistochemistry clinches the diagnosis. Rhabdomyosarcoma is positive for desmin and myogenin, whereas, CD20 and CD3 rules out lymphoma and CD99 rules out Ewings sarcoma and primitive neuroectodermal tumor which are commoner in the pediatric age group (6).

Treatment of Embryonal rhabdomyosarcoma is multimodal. Recent trends suggest that chemotherapy and radiation work well concomitantly and increase the overall disease-free survival provided embryonal rhabdomyosarcoma has not metastasized (1,8). The chemotherapy regimens currently in practice are VAC (Vincristine, Actinomycin D, Cyclophosphamide), VIE (Vincristine, Ifosfamide, Etoposide) and VAI (Vincristine, Actinomycin D, Ifosfamide). Surgery is reserved as salvage therapy, for small tumors which are non-orbital and non-parameningeal because chemotherapy and radiation alone can produce a remission rate of 91% in non-metastatic tumors (1,6,8). Our patient underwent 14 cycles of chemotherapy with definitive radiation therapy of 50 Gy following modified radical mastoidectomy with biopsy and facial nerve decompression and is tumor free for two years now. Brachytherapy has also shown promise in head and neck rhabdomyosarcoma with good loco-regional control and an acceptable side effect profile (10). Janz et al. have reported an overall survival of 59% and disease-free survival of 63% in their study of rhabdomyosarcoma of the ear (11).

## Conclusion

Early diagnosis of acute facial paralysis in childhood is essential to provide proper treatment and prevent long term morbidity in children. Embryonal rhabdomyosarcoma in childhood can present with facial paralysis and advances in the field of chemotherapy and radiation therapy have provided excellent remission rates for this tumor with a good quality of life. Surgery should be used as salvage therapy and the PET CT every year is a boon to detect recurrence which can produce a prompt treatment of the tumor.
